# Knee Muscles Composition Using Electrical Impedance Myography and Magnetic Resonance Imaging

**DOI:** 10.3390/diagnostics12092217

**Published:** 2022-09-13

**Authors:** Domenico Albano, Salvatore Gitto, Jacopo Vitale, Susan Bernareggi, Sveva Lamorte, Alberto Aliprandi, Luca Maria Sconfienza, Carmelo Messina

**Affiliations:** 1 IRCCS Istituto Ortopedico Galeazzi, 20161 Milan, Italy; 2 Dipartimento di Scienze Biomediche per la Salute, Università degli Studi di Milano, 20122 Milan, Italy; 3 Scuola di Specializzazione in Radiodiagnostica, Università di Parma, 43121 Parma, Italy; 4 Unità Operativa di Radiologia, Istituti Clinici Zucchi, 20052 Monza, Italy

**Keywords:** Skulpt Chisel^TM^, electrical impedance myography, magnetic resonance imaging, body composition, sarcopenia, muscle, knee

## Abstract

We evaluated the correlation of electrical impedance myography (EIM) measurements of knee muscles composition using Skulpt Chisel^TM^ with MRI data retrieved from muscles segmentation. A total of 140 patients (71 females, 52 ± 21 years) underwent knee MRI, EIM with Skulpt^®^, and clinical evaluation (SARC-F questionnaire). MRIs were reviewed to assess the cross-sectional area (CSA) and skeletal muscle index (SMI = CSA/height^2^) of vastus medialis, vastus lateralis, biceps, semimembranosus, and sartorius. We tested the correlations of EIM-derived parameters [body fat-percentage (BF%) and muscle quality] with total CSA, CSA of each muscle, SMI, and SARC-F scores (0–10) using Pearson correlation coefficient. We found medium negative correlation of BF% with SMI (r = −0.430, *p* < 0.001) and total CSA (r = −0.445, *p* < 0.001), particularly with biceps (r = −0.479, *p* < 0.001), sartorius (r = −0.440, *p* < 0.001), and semimembranosus (r = −0.357, *p* < 0.001). EIM-derived muscle quality showed small-to-medium positive correlation with MRI measurements, ranging from r = 0.234 of biceps (*p* = 0.006) to r = 0.302 of total CSA (*p* < 0.001), except for vastus lateralis (r = 0.014, *p* = 0.873). SARC-F scores showed small correlations with EIM and MRI data, ranging from r = −0.132 (*p* = 0.121) with EIM muscle quality to r = −0.288 (*p* = 0.001) with CSA of vastus medialis. Hence, we observed small-to-medium correlations of muscle parameters derived from Skulpt Chisel^TM^ with SARC-F scores and MRI parameters. We recommend using Skulpt Chisel^TM^ with caution for assessing knee skeletal muscles composition.

## 1. Introduction

Sarcopenia is a hot research topic due to the increasing awareness of this condition together with its rising prevalence related to world population aging. It is a disorder characterized by loss of skeletal muscle mass and strength, associated with increased morbidity and mortality related to physical disability with possible risk of falls and fractures [1]. Notably, sarcopenia has been defined as a muscle disease in the ICD-10-MC Diagnosis Code. It has been also recognized by several authors as an independent predictor of worse outcome in several conditions (i.e., tumors, chronic disorders, traumatic settings, infections) [2,3,4,5]. The loss of muscle strength occurs about 2–4 times faster than the loss of muscle mass. The increased risk of falls and fractures is also proven by the fact that the decline of lower limb muscles is greater than that of the upper limbs [6,7]. In clinical practice, the SARC-F questionnaire is routinely used for general evaluation of patient’s sarcopenic status. This is a simple poll recommended by the European Working Group on Sarcopenia in Older People to quickly recognize those sarcopenic subjects at higher risk of physical limitations and mortality [8,9,10]. Several imaging modalities are used in clinical practice and for research purposes to evaluate body composition, including dual-energy X-ray absorptiometry (DXA) that is the most used in daily routine, while computed tomography (CT) and magnetic resonance imaging (MRI) are the most accurate imaging techniques, but mostly used in research studies using a single cross-sectional image [11]. Bioelectrical impedance analysis (BIA) is an alternative non-invasive tool that uses an electrical current to measure conductance and resistance of body tissues for evaluating body composition [12]. A BIA device, namely Skulpt Chisel™, has been recently introduced to enable all subjects to assess their own muscle quality and body fat percentage (BF%) on different anatomical sites using Bluetooth™ technology to collect all measurements with smartphones [13]. This device has been introduced to estimate local and/or whole-body fat percentage. Furthermore, it provides an index of muscle quality expressed in arbitrary units, with a high muscle quality index being associated with great muscle fibers’ size, low fat content, and, in turn, high strength and a healthy muscle [14]. Good results have been published comparing the accuracy of this electrical impedance myography (EIM) device with DXA, but no data have been reported concerning the correlation of EIM results with MRI features. Therefore, we aimed at comparing the correlation of EIM measurements of knee muscles composition obtained by Skulpt Chisel^TM^ with clinical features expressed by the SARC-F scores and MRI data retrieved from muscles segmentation.

## 2. Materials and Methods

This prospective study was approved by our Institutional review board (registration number 107/int/2019 dated 20 June 2019, approved by Comitato Etico OSR). All patients signed the written informed consent to participate in this study. We matched imaging and clinical data that were then anonymized to delete any connections between data and patients’ identity according to the General Data Protection Regulation for Research Hospitals.

### 2.1. Patients Enrollment

This monocentric prospective cross-sectional study was concerned with the evaluation of a consecutive series of patients who underwent knee MRI from September 2019 to May 2021 at the IRCCS Istituto Ortopedico Galeazzi (Milan, Italy), a tertiary referral Orthopaedic Centre, for diagnostic reasons independent of the study. All patients admitted to our Institution to perform knee MRI were invited to be involved in the study to compare knee skeletal muscle mass composition obtained with MRI to that assessed through EIM using the Skulpt Chisel^TM^ device. We enrolled patients with age ≥ 18 years who were able to understand and to provide informed consent to undergo knee MRI and to participate in the study. We excluded from the study patients with: (i) clinical or psychiatric disorders that could hinder MRI or EIM performance; (ii) contraindications to MRI (such as claustrophobia, pacemakers, or neurostimulators); (iii) cancer; and (iv) pregnancy. After MRI, a radiologist performed clinical evaluation, including data collection (age, gender, height, weight, and body mass index [BMI]), the SARC-F questionnaire for quickly screen for sarcopenia, and EIM to measure BF% and muscle quality of knee muscles. The SARC-F poll includes five different evaluations (Table 1): strength, assistance in walking, rise from a chair, climb stairs, and falls. SARC-F total score range from 0 to 10 (0–2 points for each feature with 0 representing the best and 10 the worst) with a score higher than 3 being consistent with sarcopenia [9].

### 2.2. Knee MRI Protocol and Images Analysis

**Figure 1:**
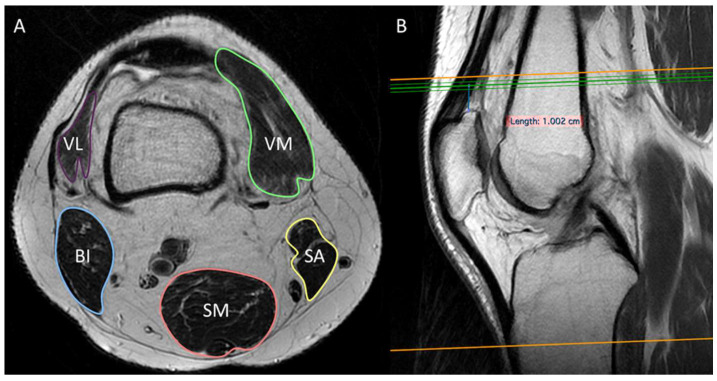
CSA and SMI. An example of manual segmentation of knee muscles on axial image (**A**) 1 cm above the patella using sagittal image as reference (**B**), with CSA values used to calculate SMI (*CSA/height*^2^). VM = vastus medialis; VL = vastus lateralis; SM = semimembranosus; BI = biceps femoris; SA = sartorius.

All knee MRIs were performed in two 1.5 T scanners (Avanto and Espree, Siemens, Erlangen, Germany, EU). Knee MRI protocol included axial T2-weighted and fat-suppressed PD-weighted, sagittal T1-weighted and fat-suppressed PD-weighted, and coronal T2-weighted and fat-suppressed PD-weighted images. A radiologist with 10 years of experience in musculoskeletal imaging and five years of experience in skeletal muscles segmentation manually drew regions of interest to assess the cross sectional area (CSA) in mm^2^ of the vastus medialis, vastus lateralis, biceps, semimembranosus, and sartorius muscles on a single axial T2-weighted image obtained 1 cm above the patella using sagittal images as reference to identify the correct axial slice (Figure 1). CSA was used to calculate the skeletal muscle index (SMI) with the following formula: *CSA/height*^2^.

### 2.3. EIM

**Figure 2:**
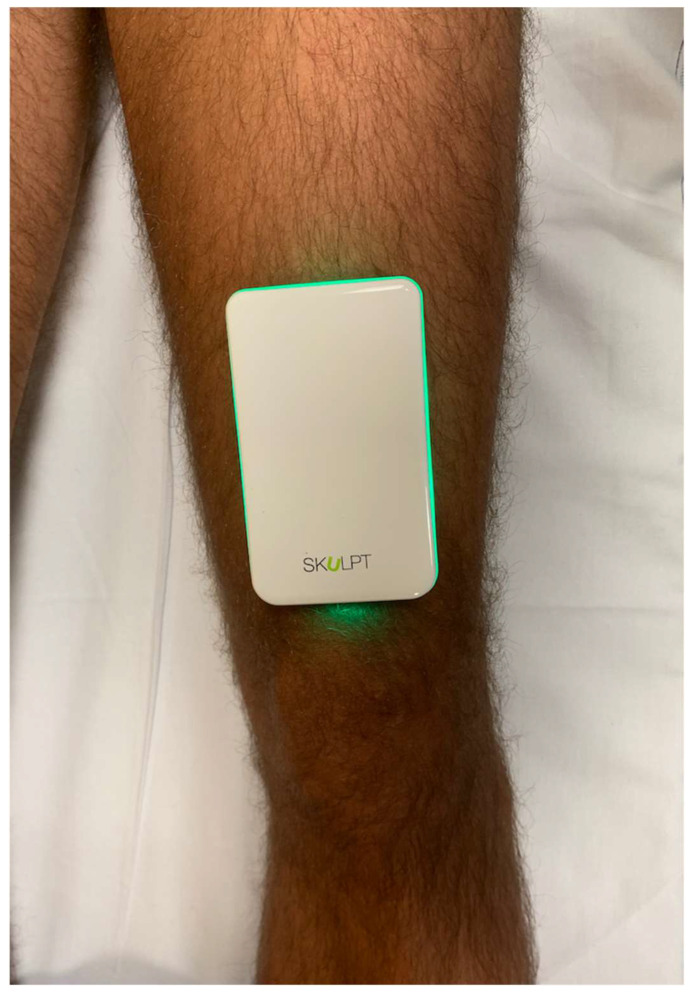
Skulpt Chisel^TM^. This figure shows how to correctly place the Skulpt EIM device on the anterior aspect of the distal third of the thigh.

BIA provides an estimation of skeletal muscle mass based on the application of an electric current across the human tissues and the measurement of current conduction [15]. This is possible due to the fact that muscles are the human tissues with the highest percentage of water. The Skulpt Chisel^TM^ (Skulpt, Inc., Boston, MA, USA) was used to measure BIA. Skulpt Chisel^TM^ is a portable device to perform EIM that, as BIA, is based on the evaluation of current conduction in human tissues to estimate body composition. The software allows to choose the muscle of interest and provides basic instructions for the use of the device. Before performing the knee measurements, a calibration was needed by applying the device to other muscles. The patient was examined while being supine on a medical bed with the knee relaxed in full extension. Normal saline was applied to the anterior aspect of the distal third of the thigh using a wipe. The electrode array was then applied to the skin and three subsequent measurements were taken. The device was connected to a smartphone with a dedicated app via Bluetooth^®^ technology. Data from triplicate measurements were averaged as a single entry in our analysis [14,16]. Data provided by the device for all patients included BF% (a measure of body fat percentage) and muscle quality, with the latter ranging from 0 (worst) to 100 (best). Figure 2 shows how we placed the device on the anterior aspect of the thigh.

### 2.4. Statistical Analysis

Categorical variables were reported as absolute values and percentages, and continuous variables were reported as means ± standard deviations or as medians and interquartile ranges according to their distribution. Normality of data was evaluated using the Shapiro–Wilk test. We analyzed the correlation between muscle quality and BF% obtained from EIM with BMI, CSA, SMI, and SARC-F scores (ranging from 0 to 10) using the Pearson correlation coefficient. We also tested the correlation of MRI data with SARC-F scores. Strength of Association was considered as follows: large (0.5 to 1.0), medium (0.3 to 0.5), or small (0.1 to 0.3). Statistical analysis was performed using SPSS^®^ software v.24 (SPSS Inc., Chicago, IL, USA). A *p* value < 0.01 was considered as statistically significant.

## 3. Results

One-hundred-forty patients were finally included in our study and were subjected to knee MRI, EIM with Skulpt^®^, and clinical evaluation including the questionnaire SARC-F. Of them, n = 71 (51%) were females and n = 69 (49%) were males. One-hundred-thirteen (80.7%) patients (66 males, 47 females) presented SARC-F scores ≤ 3, while 27 (19.3%) patients (24 females, 3 males) had SARC-F scores > 3 consistent with sarcopenic status. The mean EIM muscle quality and BF% were 50.7 ± 21.5 and 23.9 ± 8.9% in males, and 50.9 ± 21.3 and 23.9 ± 8.8% in females, respectively. Full demographic, MRI, and EIM data of our study population are summarized in Table 2.

Pearson’s correlation coefficient showed medium negative correlations of BF% obtained from EIM with SMI (r = −0.430, *p* < 0.001) and total CSA (r = −0.445, *p* < 0.001), particularly with CSA of biceps (r = −0.479, *p* < 0.001), sartorius (r = −0.440, *p* < 0.001), and semimembranosus (r = −0.357, *p* < 0.001) muscles. EIM-derived muscle quality showed small to medium positive correlations with MRI measurements, ranging from r = 0.234 of biceps (*p* = 0.006) to r = 0.302 of total CSA (*p* < 0.001), except for vastus lateralis that did not show any significant correlation (r = 0.014, *p* = 0.873). SARC-F scores showed small correlations with all EIM and MRI data, ranging from r = −0.132 (*p* = 0.121) with EIM muscle quality to r = −0.288 (*p* = 0.001) with CSA of vastus medialis muscle. All correlations of BF% and muscle quality obtained from EIM with CSA, SMI, and SARC-F scores using the Pearson correlation coefficient are reported in Table 3.

## 4. Discussion

Our main finding is the small to medium but significant correlation of muscle parameters derived from Skulpt Chisel^TM^ with SARC-F scores and MRI parameters of knee muscles composition.

This is the first study to examine the correlation of BF% and muscle quality obtained through EIM with MRI quantitative features of sarcopenia for evaluating knee muscles status. Interesting results have been published by recent studies that compared EIM with DXA. Mclester and colleagues found no significant differences (*p* > 0.05) between EIM-derived BF% and DXA, as well by BIA and skinfold measures [17]. The authors highlighted the potential of Skulpt Chisel^TM^ to estimate BF% similar to DXA, but using a fast and non-invasive device for EMI, also performing better than the widely used field method of skinfolds. Notably, their study population consisted of young healthy volunteers, while we included young and old patients subjected to knee MRI for different musculoskeletal disorders, also enrolling sarcopenic subjects. It should be considered that, despite local EIM is less sensitive to the hydration status [18], EIM evaluation can be affected by the hydration status of lean mass, given that hyperhydration may lead to underestimation of BF%, with conditions of altered hydration being more frequent in elderly [19]. These findings can partly explain our results concerning the correlations between Skulpt Chisel^TM^ and MRI quantitative parameters for assessing knee muscles composition. Of note, Czeck et al. reported significantly lower regional BF% measured by Skulpt Chisel^TM^ than DXA in the upper arms, right leg, and trunk, although no significant differences were reported in total BF% [20]. Their results may partly justify our medium correlation between BF% and MRI measurements, since Skulpt Chisel^TM^ has been shown to be accurate for evaluating global BF% also by other authors [17,21]. At any rate, it should be taken into account that local BF% obtained with EIM seems to be mostly related to subcutaneous fat, as recently highlighted by Longo et al. [14]. Furthermore, limits of this device have been underlined by Wells et al. who found significant differences in BF% when comparing EIM data with seven-site skinfold and hydrostatic weighing BF% estimates (*p* < 0.05) [13].

In our study, we used the CSA and SMI as imaging features to evaluate the correlation of Skulpt Chisel^TM^ parameters with those of MRI. The accuracy of MRI in assessing CSA on cross-sectional images has been shown to be almost identical to that of the reference standard, namely CT (up to r = 0.99) [22,23]. In this setting, CSA can be applied to calculate the SMI with cut-off points established by previous papers to identify sarcopenic subjects [24]. As a matter of fact, measurements obtained from MRI axial images are highly accurate for evaluating body composition, while DXA allows obtaining an estimation of total body lean mass [11,23,25]. Someone may argue that the CSA and SMI are not direct parameters of muscle quality, such as inter- and intra-muscular fat tissue infiltration [26]. This could explain the limited correlation with EIM-derived features, but, as abovementioned, these MRI parameters are well-established and recognized as accurate methods to differentiate sarcopenic from non-sarcopenic patients. A note should be made concerning the vastus lateralis muscle. Its distal myotendinous junction is slightly proximal to that of the vastus medialis, thereby making its muscle belly scarcely or, in some cases, not visible 1 cm above the patella (see Figure 1). Thus, its CSA is too variable from one patient to another, thus making this measure not reliable for assessing the sarcopenic status at this level of the thigh. This is the reason why the CSA of vastus lateralis showed the worst correlations with the other investigated parameters.

Lastly, concerning clinical features of our series, we observed small correlations of SARC-F scores with BF%, EIM-derived muscle quality, CSA, and SMI. However, it must be considered that SARC-F poll is a useful clinical questionnaire for evaluating low muscle strength and physical performance, but with well-established limitations, particularly for screening patients with sarcopenia [1,27,28].

A limitation of our study is that manual segmentation of knee muscles was done by a single experienced reviewer, but it has been proven that these measurements are strongly reliable and reproducible, being widely used for research purposes [11,25,29]. The same can be stated for EIM-derived parameters obtained with Skulpt Chisel™, with previous studies that demonstrated the high reliability of these measurements [30]. Another limitation is the absence of a clear diagnosis of sarcopenia, which is generally done through whole body DXA [31,32], to be used as reference standard to assess the diagnostic performance of EIM-derived parameters to differentiate sarcopenic and non-sarcopenic patients, which might be the focus of further studies. Then, we performed a comparison of mere morphologic data, while these findings need to be compared also with functional parameters, given that muscle strength and mass modify over time at different speeds [33].

## 5. Conclusions

In conclusion, EIM-derived parameters obtained by Skulpt Chisel^TM^ showed small to moderate degrees of correlation with SARC-F scores and MRI features used for evaluating muscles mass status around the knee. We recommend cautiously using this device for assessing skeletal muscle composition around the knee. Further studies in this setting are warranted. In the future, it should be investigated the association of EIM-derived parameters with other morphologic features derived from CT and DXA, but also with functional data concerning muscle strength and performance. Moreover, it should be investigated the predictive role of EIM-derived features in specific musculoskeletal disorders (e.g., neuromuscular diseases), risk of fall and fractures, and other conditions in which sarcopenia has shown to be a negative predictor such as cancers, surgery, and chronic disorders.

## Figures and Tables

**Table 1:** SARC-F questionnaire.

Feature	Question	Answer
*Strength*	How much difficulty do you have in lifting and carrying 10 lb?	None = 0 Some = 1 A lot or unable = 2
*Assistance in walking*	How much difficulty do you have walking across a room?	None = 0 Some = 1 A lot, use aids, or unable = 2
*Rise from a chair*	How much difficulty do you have transferring from a chair or bed?	None = 0 Some = 1 A lot, or unable without help = 2
*Climb stairs*	How much difficulty do you have climbing a flight of 10 stairs?	None = 0 Some = 1 A lot, or unable = 2
*Falls*	How many times have you fallen in the past year?	None = 0 1–3 falls = 1 ≥4 falls = 2

**Table 2:** Demographic, MRI, and EIM data of 140 patients prospectively enrolled in our study.

**Age**	52 ± 21 years (range 18–89)
**Weight**	76 ± 18 kg (range 51–180)
**Height**	169.2 ± 10.1 cm (range 145–195)
**BMI**	26.5 ± 5.8 (range 15.8–62.3)
**SMI**	894 ± 284.3 (range 491.5–1623.7)
**Muscle quality**	50.9 ± 21.3 (range 10–96)
**BF%**	23.9 ± 8.8% (range 8.3–51.6)
**SARC-F**	1 (IQR = 0–3, range 0–10)

BMI = body mass index; SMI = skeletal muscle index; BF% = Body fat percentage IQR = interquartile range (1–3).

**Table 3:** Correlations of SARC-F scores, EIM, and MRI data using the Pearson correlation coefficient.

		BF%	M.quality	SARC-F	CSA	SMI	VM	VL	Biceps	SM
**BF%**	r	1	−0.396 **	0.283 **	−0.445 **	−0.430 **	−0.337 **	−0.135	−0.479 **	−0.357 **
	*p*		<0.001	0.001	<0.001	<0.001	<0.001	0.12	<0.001	<0.001
**M.quality**	r	−0.396 **	1	−0.132	0.302 **	0.283 **	0.245 **	0.014	0.234 **	0.282 **
	*p*	<0.001		0.121	<0.001	0.002	0.003	0.873	0.006	0.001
**SARC-F**	r	0.283 **	−0.132	1	−0.241 **	−0.208 *	−0.288 **	−0.198 *	−0.225 **	−0.122
	*p*	0.001	0.121		0.004	0.021	0.001	0.022	0.008	0.152
**CSA**	r	−0.445 **	0.302 **	−0.241 **	1	0.952 **	0.862 **	0.528 **	0.806 **	0.884 **
	*p*	<0.001	<0.001	0.004		<0.001	<0.001	<0.001	<0.001	<0.001
**SMI**	r	−0.430 **	0.283 **	−0.208 *	0.952 **	1	0.807 **	0.509 **	0.763 **	0.871 **
	*p*	<0.001	0.002	0.021	<0.001		<0.001	<0.001	<0.001	<0.001
**VM**	r	−0.337 **	0.245 **	−0.288 **	0.862 **	0.807 **	1	0.466 **	0.564 **	0.612 **
	*p*	<0.001	0.003	0.001	<0.001	<0.001		<0.001	<0.001	<0.001
**VL**	r	−0.135	0.014	−0.198 *	0.528 **	0.509 **	0.466 **	1	0.382 **	0.414 **
	*p*	0.12	0.873	0.022	<0.001	<0.001	<0.001		<0.001	<0.001
**Biceps**	r	−0.479 **	0.234 **	−0.225 **	0.806 **	0.763 **	0.564 **	0.382 **	1	0.629 **
	*p*	<0.001	0.006	0.008	<0.001	<0.001	<0.001	<0.001		<0.001
**SM**	r	−0.357 **	0.282 **	−0.122	0.884 **	0.871 **	0.612 **	0.414 **	0.629 **	1
	*p*	<0.001	0.001	0.152	<0.001	<0.001	<0.001	<0.001	<0.001	
**Sartorius**	r	−0.440 **	0.306 **	−0.086	0.757 **	0.707 **	0.543 **	0.275 **	0.683 **	0.649 **
	*p*	<0.001	<0.001	0.313	<0.001	<0.001	<0.001	0.001	<0.001	<0.001

M.quality = muscle quality obtained from EIM; BF% = body fat percentage obtained from EIM; = CSA = total cross-sectional area of knee muscles; SMI = skeletal muscle index; VM = CSA of vastus medialis; VL = CSA of vastus lateralis; SM = CSA of semimembranosus; r = Pearson correlation coefficient; *p* = significance; ** = significant values with *p* < 0.01; * = significant values with *p* < 0.05.

## Data Availability

All data are fully available upon reasonable request. The corresponding author should be contacted if someone wants to request the data.
